# The impact of Artificial Intelligence in academia: Views of Turkish academics on ChatGPT

**DOI:** 10.1016/j.heliyon.2023.e19688

**Published:** 2023-09-01

**Authors:** Tuba Livberber, Süheyla Ayvaz

**Affiliations:** a Department of Journalism, Faculty of Communication, University of Akdeniz, Antalya, Turkey; b Department of Advertising, Faculty of Communication, University of Selcuk, Konya, Turkey

**Keywords:** Academia, Artificial intelligence, Human-AI collaboration, Learning & teaching, Machine learning

## Abstract

In the past decade, Artificial Intelligence (AI) and machine learning technologies have become increasingly prevalent in the academic world. This growing trend has led to debates about the impact of these technologies on academia. The purpose of this article is to examine the impact of ChatGPT, an AI and machine learning technology, in the academic field and to determine academics' perceptions of it. To achieve this goal, in-depth interviews were conducted with 10 academics, and their views on the subject were analyzed. It is seen that academics believe that ChatGPT will play a helpful role as a tool in scientific research and educational processes and can serve as an inspiration for new topics and research areas. Despite these advantages, academics also have ethical concerns, such as plagiarism and misinformation. The study found that ChatGPT is viewed positively as a useful tool in scientific research and education, but ethical concerns such as plagiarism and misinformation need to be addressed.

## 1 Introduction

Artificial Intelligence (AI) and machine learning technologies, which hold an important place among developing technologies, have rapidly developed and started to be used in many fields in recent years [1,2]. These technologies can create outputs similar to those produced by humans and can significantly reduce people's energy and time [[1], [2], [3], [4]]. The concept of AI generally encompasses a set of technologies and techniques related to the ability of computer systems to perform tasks that require human intelligence [5]. In other words, AI is the simulation of human intelligence in machines programmed to think and act like humans [2]. AI technologies appear as examples of a kind of ‘datafication’ process that affects society as a whole [6]. Among the sub-areas of these technologies are machine learning, supervised learning, unsupervised learning, natural language generation and natural language processing (NLP) [7].

One of the types of chatbots that uses AI to understand user inputs in a natural, human-like way and to respond to them is the AI chatbot. AI chatbots are designed to chat with humans using NLP to understand and respond to users' words and intentions [2]. One of these chatbots is the text-based chatbot. ChatGPT (Chat Generative Pretrained Transformer 3.5) is a text-based large language model trained by OpenAI, a San Francisco-based initiative founded in 2015 by Elon Musk, Sam Altman, Greg Brockman, Ilya Sutskever, Wojciech Zaremba, and John Schulman [8]. ChatGPT is part of the GPT series of models released by OpenAI. The first GPT, GPT-1, was introduced in 2018 and has been used and explored in many articles, patents, and services around the world. In 2019, OpenAI introduced GPT-2, but the full data of the model was not published due to its potential misuse by many people. In 2020, OpenAI introduced GPT-3.5, which is the largest and most advanced language model of OpenAI. ChatGPT is a version of the GPT-3.5 model and is a chatbot trained by OpenAI. In this sense, ChatGPT is a language model that can learn from a large amount of data and provide responses that are suitable for human language [8].

ChatGPT is widely used in various fields in academia. The use of AI technologies such as ChatGPT in areas such as academic research, education, and access to knowledge has also been the subject of studies. ChatGPT is used for various purposes in academia, including language translation, document summarization, inference, question-answering systems, and language modeling [8]. Therefore, understanding the impact of chatbots such as ChatGPT on academia and the perception of academics towards these technologies is crucial. However, scientific articles on the impact of ChatGPT on academia have generally remained at the theoretical level, and discussions have aroused on the responses given by ChatGPT itself [[9], [10], [11]]. In this context, it is difficult to come across with a study on academics' experiences. Therefore, this article examines the impact of ChatGPT in the academic world and indicates how academics perceive this impact. The study reveals how ChatGPT is used in academia, potential benefits and drawbacks it offers, what the attitudes and opinions of academics are towards ChatGPT and its use in academia. Additionally, the study also reveals the current status of the applications of ChatGPT technology in academia and their thoughts on future developments.

## 2 ChatGPT and academic universe

ChatGPT, after being released to the public on November 30th, 2022, attracted the attention of over a million users in just one week and received significant media coverage [[12], [13], [14]]. This success demonstrates that ChatGPT is one of the most exciting developments in the field of AI [15]. ChatGPT is considered the most advanced language model ever created [3] and is the strongest among chatbots [16]. The future of ChatGPT is also promising. Some experts believe that it could replace Google and become the world's best search engine within a few years [17]. As Manjoo [18] expressed, machines' writing ability is developing in a frighteningly impressive way. ChatGPT is a significant pioneer in this field and can now be used not only for writing computer code but also for different areas such as home decoration and marketing idea generation [17].

ChatGPT, designed to make the interaction between humans and AI more natural, can generate meaningful answers to given questions [[19], [20], [21], [22]]. Additionally, ChatGPT is a highly sophisticated chatbot that can perform more complex tasks, such as guiding productivity problems, as well as simple tasks such as answering basic questions and writing thank-you letters [21]. ChatGPT can interact with users in a natural and intuitive way, making the use of AI technology more widespread [3]. Another advantage of ChatGPT is its ability to be used as a search engine. This feature allows users' questions to be answered with contextually relevant information. Studies conducted by Aljanabi et al. [23], Hammad [24], and O'Connor and ChatGPT [2] have shown that ChatGPT can understand the intentions behind a question and provide users with the information they need more quickly and effectively.

The use of ChatGPT in various fields has significantly increased in recent times. This feature allows users to access the information they need quickly and easily. The combination of AI technologies enhances ChatGPT's natural language generation and provides users with a smoother and more intuitive experience [15]. Additionally, the integration of computer vision and robotic technologies with ChatGPT will enable the development of intelligent and conversation-based AI systems [15]. This can bring many innovations in the way we interact with technology. According to Mijwil et al. [25] better training algorithms and larger datasets are critical to continuously improving language model performance, which is essential for the future of ChatGPT. In this way, new and innovative applications that require the ability to analyze and understand large amounts of information in areas such as healthcare and finance can be developed. Another possibility for ChatGPT is the potential for more personalization and customization through learning from user interactions and individual preferences [15]. As ChatGPT interacts with users, it can learn their language, tone, and style to produce more personalized and accurate responses. This increased level of personalization can also improve customer service and education [15].

ChatGPT has emerged as a tool that can be used in the preparation of academic/scientific texts, along with its impact that has been featured in international news [26]. The model has the ability to understand and respond to various natural language inputs, having been trained on a large corpus of text data [27,28]. In fact, in some scientific studies, information provided by ChatGPT has been used as scientific knowledge [25,29]. ChatGPT can provide significant time and effort savings to researchers by helping with tasks such as creating article summaries, identifying key points, and providing quotations [23]. Additionally, ChatGPT can also take on various tasks for academic texts. For example, it can create texts for various academic document types including research papers, essays, and theses. It can also help authors improve their work by providing feedback on grammar, style, and consistency. ChatGPT can become an effective tool for teaching and learning as well [1]. For instance, it can assist students in understanding and summarizing difficult texts and can also generate prompts for writing assignments [1].

Another advantage of ChatGPT is its ability to be a powerful tool for content creation. The model can generate new texts, which can facilitate the content creation process [2,23]. Le [30] suggests that academics could collaborate with AI to make their writing tasks easier. The idea is to give detailed clues to the AI so that it can create separate paragraphs and then combine them. In addition, it is also noted that ChatGPT can write an article [31]. ChatGPT's writing ability has amazed people with its mastery in complex tasks and ease of use. However, the widespread use of ChatGPT may raise concerns among some academics about becoming unemployed in the future [32]. In this context, it is believed that the progress of machine learning and AI technologies may lead to changes in people's roles and ways of doing business in the labor market.

However, many researchers point out that ChatGPT cannot replace human intelligence and creativity and does not have the ability to conduct original scientific research on its own [33,34]. Nguyen [1] also states that ChatGPT lacks systematic reasoning ability for multi-paragraph and precise information required for academic papers. In addition, it is emphasized that the output generated by ChatGPT is not always 100% accurate and needs to be verified for accuracy by the user [1,23]. Hammad [24] emphasizes that ChatGPT still does not fully understand the nuances of human language and social media communication, and therefore may not always provide the most accurate or useful information. Additionally, ChatGPT's critical thinking ability is limited and it does not have expertise in a specific scientific field required for conducting scientific research [33,35]. In the study conducted by Methnani et al. (2023), the capability of ChatGPT, to calculate sample size for sports sciences and sports medicine research was investigated. Through the analysis of four distinct studies, it was observed that ChatGPT was capable of correctly calculating the sample size for a randomized controlled trial when provided with the necessary data. However, it failed in the remaining three instances. Intriguingly, it was noted that the reuse of the same prompt resulted in a different sample size from the model. These results, despite the potential of AI, serve as a caution for scientists to exercise diligence while utilizing these tools [36].

There is concern that ChatGPT may not reflect the cultural, ethical, and societal values that academics prioritize [8]. Although ChatGPT is quite successful in processing, distilling, and presenting information verbally or in writing, it is not conscious and lacks self-awareness [34]. ChatGPT has not yet reached the necessary competence to truly generate creative or original ideas. As noted by Hammad [24], ChatGPT can currently only conduct research based on statistical processes and probabilities. Another disadvantage of ChatGPT is its limited scope and depth of knowledge [34]. Unlike search engines, ChatGPT cannot scan the web for information on current events, and its knowledge is limited to what it learned before 2021 [37,38].

As with any rapidly advancing technology, it is important to consider the potential ethical and societal impacts of ChatGPT. Concerns such as privacy and employment effects are among the issues that need to be carefully evaluated as these technologies continue to develop. Therefore, it is important to evaluate the ethical implications of ChatGPT and to develop and use it responsibly and ethically [15]. For example, in random tests conducted with plagiarism detection software, ChatGPT's work was not detected [3] and Marcus and Davis [39] declared that ChatGPT “rambles fluently” and “is not a reliable interpreter of the world."

## 3 Method

This research aims to discuss the potential impact of ChatGPT technology on the academic world, from the perspective of the experiences of academics. In this context, the research aims to understand how academics interpret the effects of AI on scientific research and the education process. To achieve this goal, we have set two specific objectives:

1. To collect the views of academics on ChatGPT technology.
2. To explore the impact of ChatGPT on the professional lives of academics.

Research questions:

RQ1 How is ChatGPT integrated into academic research?
RQ2 How is ChatGPT integrated into the education process?
RQ3 What are the advantages and disadvantages of ChatGPT?
RQ4 How is ChatGPT evaluated in terms of academic ethics?
RQ5 What impact will ChatGPT have on the future of the academic world?

The preferred research approach in this study is qualitative research, which is used when a problem or topic needs to be explored and existing theories are partial or inadequate in explaining the complexity of the problem. The purpose of qualitative research is not to generalize information, but to make specifically specific explanations [40]. The history of AI becoming a useable technology in academic research starts with the emergence of ChatGPT. The theoretical foundation regarding the impact of ChatGPT on academic life is still in its initial stages, as this technology has not yet even completed its first year. The phenomenological research design chosen aims to gain a deep understanding of the nature of an experience based on the fundamental question of “what is this experience like?” [41]. In this context, the impact of ChatGPT on academic life is discussed based on the experiences of academics in this research.

In this study, data were collected using semi-structured interviews through the phenomenology design within the qualitative paradigm, and thematic analyses were conducted using Maxqda qualitative data analysis software. The research group was determined through purposive sampling based on two criteria: 1) being an academic, and 2) having experience with ChatGPT. Academics with ChatGPT experience around us and those who had commented on ChatGPT in various newspaper articles through Twitter were identified. In February 2023, 18 academics from Turkey were invited to volunteer for the study, but only 12 responded to our invitation. Saturation was reached after 10 interviews, and responses started to repeat. In March 2023, the interviews were completed and the data were extracted. The sample size in qualitative research depends on the research design. The participant group in phenomenology design studies generally consists of 3–10 individuals [42]. Participants were anonymized by assigning C1, C2…C10 names to each data transcription file. The second author opened a project file in the Maxqda program and uploaded the transcripts to a project file.

The majority of the participants are in their 30s (6), have a PHD degree (5), and are male (7). Demographic information of the participants is presented in Table 1:

**Table 1:** Demographic data of participants.

Demographics		Sum
Gender	Female	3
	Male	7
Age	20–29	2
	30–39	6
	40–49	1
	50–59	0
	60–69	1
Education	Bachelor's Degree	2
	Master's Degree	3
	PhD	5

Looking at the academic backgrounds of the participants, the majority works at a state university (7), and there is diversity in terms of academic titles, with half of them not yet having completed ten years in the academic profession (See: Table 2):

**Table 2:** Academic background data of the participants.

Academic Background		Sum
University Type	Government University	7
	Private University	3
Academic Title	Research Assistant	2
	Lecturer	3
	Assistant Professor	2
	Associate Professor	2
	Professor	1
Academic Experience	1–9 years	5
	10–19 years	4
	20 years +	1

**Fig. 1:**
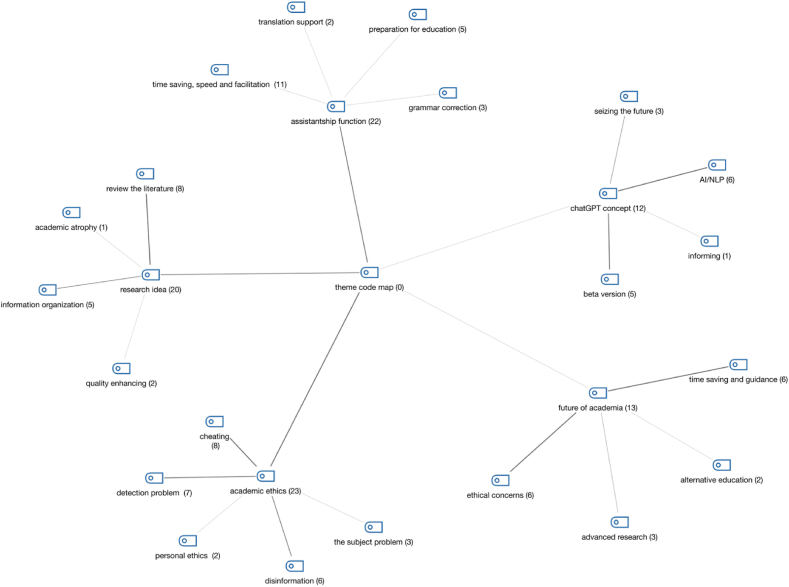
“Theme code map”.

Two documents randomly selected from the decoded ones were independently coded by two authors. The first author manually coded the printouts of the selected two documents, whereas the second author coded the documents in the Maxqda software. After the coding was completed, the authors held an online meeting. During this meeting, they discussed the sections and codes from the two codings that matched and the ones that didn't, in an attempt to reach a consensus among the coders. Although both researchers didn't use identical concepts in themes and subcategories such as ChatGPT, the future of ChatGPT, ethical concerns about ChatGPT, and ChatGPT serving as an auxiliary tool (for example the first author labeled the theme on ethics as “ethical concerns”, whereas the second author labeled it as “academic ethics”), they were seen to have done similar codings. Moreover, differences were identified in the subcategories and sub-codes of the themes of the two researchers. For instance, while one grouped codes such as time-saving and literature scanning under a single theme, the other evaluated these two codes under separate themes. To resolve these conceptual differences and subcode/category discrepancies, both researchers re-read the participant statements in the selected decoded document and jointly decided on the appropriate code and theme. In some cases, the codes of the first author were adopted, in some cases, the codes of the second author were adopted, and sometimes a new code concept emerged. As a result of this meeting, which lasted approximately 4 h, a theme code map was largely determined. Then, the second author coded the remaining decoded documents in Maxqda based on this code map. During this coding, the second author sometimes encountered new codes (for example grammar correction). After completing the initial coding, the author evaluated the themes and categories according to the research question during the second and third readings and brought together the common codes. Then, another online meeting was held with the first author, during which the authors discussed the codings over approximately 3 h. The second author opened the Maxqda project file for discussion by sharing the screen with the first author. As a result of the discussion, there was a disagreement about dividing the ethical theme into transparency and ethics. However, considering the data density and semantic level, it was ultimately decided that these two themes could be combined. In the final stage, another meeting was held with a selected participant, and the correctness of the codings was questioned. Thus, the reliability and validity of the research were strengthened. The theme map created as a result of these processes is presented in the following Fig. 1:

AI: Artificial Intelligence.
NLP: Natural Language Processing.

According to the theme code map, the most frequent interpretation regarding academics' experiences with ChatGPT is related to its idea generation and assistantship functions. Based on the frequency indicators of these two themes and their codes, the most significant feature of ChatGPT is its role as a tool to assist academic productivity. However, the third frequent interpretation is related to academic ethics. In fact, considering both this theme and the ethical concerns in the following theme, ChatGPT's second most important feature for academics is that it raises ethical concerns.

## 4 Results

### 4.1 ChatGPT concept

This theme was constructed to reveal what the participants knew about the ChatGPT technology at the center of the experience. Although this theme does not have the potential to directly answer the research questions, it serves as an introduction to the analysis.

Examining the nature of ChatGPT will offer insights into how it is perceived in the academic universe. In this regard, how academics position ChatGPT is crucial. Academics approach ChatGPT from four perspectives. The first is that ChatGPT is at the center of the current stage of NLP models as an anthropomorphic AI technology similar to humans. However, related to this, the second aspect is that ChatGPT is still in its early stages of development and in beta phase. Many academics believe that despite being in beta, ChatGPT has a great potential and will revolutionize life. According to the participants, the third point is that ChatGPT will shape the future. ChatGPT is considered to be a promising invention that will spread and create innovations in many fields and professions, transforming them. In addition, ChatGPT is generally interpreted by participants as a positive technological development that will bring about many changes initially:

The emergence of ChatGPT is one of the results of the effective computational technologies that have been achieved in the 2020s. It has emerged as a system that can produce innovative results by effectively processing a large number of complex data. ChatGPT has attracted so much attention because it can derive new data from the data it uses in a way that is closest to human perception and understanding. In my opinion, ChatGPT is a modified version of the advanced, creative Deep Learning-based AI infrastructure called GPT, which OpenAI has been developing for some time. Similarly, the company had previously developed a system called Dall-E using this infrastructure. After ChatGPT, I think we will see many different, alternative (and of course, impactful) GPT applications (C7).

In today's world where AI technologies are rapidly developing, I believe that such applications are inevitable and expected. Therefore, it is important to follow current developments, try to understand them, and analyze these developments in terms of society and technology. ChatGPT is an important step in understanding the lifestyles of the future, especially in fields such as philosophy, art, and communication, which are frequently discussed. Science fiction themes that are the subject of literature and cinema are beginning to create today's reality. Considering the interest of our age in technology and the steps taken in this direction, ChatGPT is not a surprising development (C3).

### 4.2 Assistantship function

This theme answers the first three of our research questions. Participants' views on how ChatGPT is integrated into academic research and the educational process (RQ1 and RQ2) and its advantages and disadvantages (RQ3) were evaluated under this theme.

ChatGPT has a functional meaning for academics. ChatGPT provides advantages to academics by supporting them in many aspects of education and academic production processes. In this regard, the participants describe ChatGPT directly as an “assistant-like” tool or with similar expressions:

ChatGPT is functional for academics in many areas such as finding topics for academic articles, designing article structures, accessing literature, making both semantic and formal corrections and edits on the text. Additionally, it has started to be used in academic life to expand course content and provide new perspectives (C10).

Participants state that the integration of ChatGPT into the academic process creates a significant advantage, especially in terms of saving time. ChatGPT provides time economy in the academic production process by shortening time-consuming processes in accessing and organizing information related to the research area, enabling academics to reach and organize information quickly. Participants recognize that ChatGPT speeds up and facilitates academics' work. Speed and convenience particularly emerge in the literature review stage, which requires a significant time investment at the beginning of the academic production process. According to the participants, ChatGPT makes it easier to examine the accumulated literature, which is now almost ubiquitous in almost every field, and to benefit from more sources. Integrating ChatGPT into academic work helps to scan a large body of literature quickly and more easily, while at the same time enabling academics to overcome language barriers. It has the advantage of facilitating the review of literature worldwide. ChatGPT can facilitate many aspects of scientific writing, such as organizing and reviewing the content and format of scientific texts, identifying and correcting errors, and in this way, can speed up the work of academics:

I don't know how it is integrated into academic studies since I am not conducting an academic study, but I believe that ChatGPT's ability to quickly access data, translate, paraphrase, etc. will also facilitate the work of the academic community (C4).

Finally, the convenience provided by ChatGPT in academic processes also carries the risk of weakening the ability to conduct academic research. ChatGPT has the disadvantage of weakening academic production skills:

The increasing ease of accessing information through these technologies can lead to the erosion of research skills for both students and academics (C8).

### 4.3 Research idea

This theme answers two of our research questions. Participants' views on how ChatGPT is integrated into academic research (RQ1) and its advantages and disadvantages (RQ3) were evaluated under this theme.

Academics state that ChatGPT can not only serve as an auxiliary tool in the academic production process by being integrated into academic work, but also provide advantages in the idea generation process. According to the participants, ChatGPT is a remarkably advantageous technology. It aids in formulating research ideas, keeping up with field-specific literature, becoming cognizant of novel approaches, gaining fresh perspectives, identifying key concepts that shape the essence of a research idea, and achieving excellence in structuring the research idea within the study. It is considered a useful tool in terms of achieving quality in the organization of research ideas within a study. In addition, it has contributions to the creative process in constructing the research idea and transforming the idea into meaningful knowledge. However, it is emphasized that ChatGPT will only provide unprocessed information related to the research idea, and the creativity process involving the construction of this information is dependent on human beings:

Especially in my studies related to social sciences, I use ChatGPT to generate ideas when I experience a blockage. In some cases, if there are sources that I have not been able to reach, I consider them. One of the general difficulties of academics is that we often miss alternative perspectives when we focus on a topic. At this point, ChatGPT can provide us with alternative approaches … The use of ChatGPT is highly beneficial and suitable for improving the quality of the study (C9).

Especially in the field of social sciences, it is a functional area to follow current developments and to demonstrate their impact on society. Evaluating an application such as ChatGPT within this framework and identifying its possible consequences is one of the examination subjects of academia. Therefore, I believe that this development is important in terms of providing a new and notable research topic within the scope of academic research … However, on the other hand, the claim that the application has a kind of academic role in producing, processing, and conveying information is far from reality at this stage. Seeing the processed and reconstructed information as a primary extension of a profession means taking a broader perspective. In this case, Google can also play a professional role as a comprehensive library or a book. However, any development in which humans are not involved or disregarded will be limited for these tools produced by human hands (C3).

### 4.4 Academic ethics

This theme answers two of our research questions. Participants' views on the advantages and disadvantages of ChatGPT (RQ3) and the effect of ChatGPT on academic ethics (RQ4) were evaluated under this theme.

ChatGPT and similar technologies that generate information in a relatively meaningful textual form have the potential to create ethical violations, which is a serious cause for concern. Ethical problems are related to both academic knowledge production processes and academic education-teaching processes. On the one hand, the production of scientific articles with ChatGPT and, on the other hand, the creation of student assignments with ChatGPT raise concerns. Currently, many investigation files regarding ethical rule violations in academic publications are still being opened. In addition, the production of copy-paste assignments by students via the internet also creates serious plagiarism problems. Therefore, it is possible to talk about two types of academic cheating:

… some academics or academic candidates are able to add the writings generated by ChatGPT to their work as if it were their own produced knowledge, and there are even some who directly have articles, papers, and even theses written by ChatGPT (C6). I have some concerns about plagiarism issues, it may lead to cheating issues among students when writing academic essay or doing their assignment and it will be hard for academicians or lecturers to identify cheating issues (C5).

The use of ChatGPT in academic production processes raises the issue of who owns the information. This problem can be expressed as the knowledge/subject dichotomy or the subject problem. It suggests that the limits of adding the information produced by ChatGPT verbatim or indirectly to scientific texts should be seriously discussed from an ethical perspective. While questioning the subject of knowledge, the value attributed to scientific knowledge produced by ChatGPT also undermines ethical boundaries. While plagiarism can be solved through the referencing system, it also assigns ownership to the information. According to the participants, there is a need for legal regulations that clearly define the role of ChatGPT in the production of scientific knowledge and its indication through citation. However, the problem of detecting plagiarism arises here. Various technological developments are needed to detect direct or indirect use of ChatGPT. The current plagiarism detection programs may not have sufficient competence in this regard for some academics. On the other hand, for some academics, necessary plagiarism detection programs have been developed that reveal the potential misuse of ChatGPT within the scope of plagiarism. It should be noted that ChatGPT is an evolving technology, and such plagiarism detection programs will also need to continuously develop in parallel. However, it is not only plagiarism programs that are effective in overcoming ethical problems arising from plagiarism, but also individuals' personal ethical understanding. Participants emphasize that ethical problems are ultimately related to individual ethics. The possibility of plagiarism from ChatGPT creates a suspicious situation for both students and academics in terms of “scoring”. The difficulties in detecting plagiarism will raise questions about the validity of the grades given to student assignments and the points accumulated by academics in their professional advancement:

ChatGPT can be used to simplify our work, but it should be used within ethical boundaries, as I have heard that in plagiarism detection programs like Turnitin, it cannot identify text written by ChatGPT and its derivatives as they are considered original (C4) … However, software has already been developed to detect these (C10).

Individual ethical understanding is crucial in this matter. The use of ChatGPT can be highly beneficial and appropriate to enhance the quality of a study. Just as it is normal and healthy to take ideas from others, using ChatGPT to some extent is equally legitimate. However, relying heavily on ChatGPT outputs in a study is akin to plagiarism (C9).

Not being able to determine exactly how the student assignments are completed can create some difficulties in grading assignments … Although I think that it would be possible to detect plagiarism rates more accurately in academic activities such as thesis, articles, and book chapters, in a country where there are no publications, rectorates, and plagiarizing professors are treated the same way and nothing happens to them, even if the truth is revealed in all its nakedness, there cannot be an ethical development (C8).

As it questions the validity of academic progress, it also causes doubt on the “accuracy” and “impartiality” of academic knowledge. As ChatGPT has also stated, “While we have safeguards in place, the system may occasionally generate incorrect or misleading information and produce offensive or biased content”. In this respect, disinformation makes the information produced by ChatGPT ethically questionable. Academics are developing various strategies to determine whether the information obtained from ChatGPT is disinformation or not. For example, information obtained from another information source such as search engines is attempted to be verified. The possibility of suspicious and biased information from ChatGPT makes academics cautious about this technology:

However, I believe that for now, it is more prudent to remain in the gray area, recognizing that ChatGPT and similar technologies may become tools for malicious purposes such as propaganda, polarization, fake news, and disinformation in the future (C8). I can obtain information from ChatGPT rapidly, which would otherwise require hours of exploration on search engines like Google. On the other hand, I verify the information obtained from ChatGPT by researching it on search engines (C6).

### 4.5 Future of academia

This theme answers one of our research questions. Participants' views on the impact of ChatGPT on academic future (RQ5) were assessed.

When questioning what the future of academic life will be like in a world with ChatGPT, it can be seen that assessments regarding the current situation also encompass the future. Ethical concerns, in particular, are frequently mentioned by academics regarding the future of academia. There is a particular emphasis on determining the role of ChatGPT and academics in producing scientific knowledge, developing plagiarism detection software, and clarifying the ownership of information. If these types of problems are solved, it is noted that ChatGPT has a valuable potential in terms of finding answers to many questions in the academic future. At this point, there is essentially a binary future for academic life with ChatGPT. On the one hand, the positive use of ChatGPT, which everyone can benefit depending on their personal ethical understanding, may be possible. On the other hand; there is the possibility of misusing such as pushing people towards convenience and using it for their personal interests.

It may create problems in some cases where there is direct ownership of information or claiming ownership of something that does not belong to us. Therefore, it is important to develop new tools similar to plagiarism detection reports to reveal the true subject of the information and research. ChatGPT and other similar AI applications are becoming subjects in themselves, taking the place of reality with their ability to access, process, and replicate information. Therefore, having regulatory mechanisms in academic production is necessary to establish ethical values and prevent production based on malicious intent (C3).

If used within the framework of ethical values by moral individuals, like everything else, it will improve us all. However, if someone uses a program like ChatGPT to write articles or papers just to get a title or pass a course, it will be an unethical act and unacceptable. I hope it will be used to facilitate research and contribute to finding answers to many questions. But at the same time, I am afraid that such a powerful tool may not be used positively in the hands of someone who is inclined to laziness and self-interest. Therefore, I have concerns that academics or candidates may not use this system within ethical boundaries (C4).

The second topic regarding the academic future is about the benefits that ChatGPT can provide. This is related to the fact that, just like in its assistant function, ChatGPT can guide academics and provide significant time and energy savings. It will contribute to academics in every aspect, from literature review to producing scientific texts and formal stages. It is believed that ChatGPT will provide an advantage to academic life, especially in terms of time savings, in the future. However, it is also mentioned that the impact of ChatGPT on traditional education and academic processes will be limited. In this sense, ChatGPT has an indirect effect on academic life. According to academicians, the opportunities provided by ChatGPT such as speed, guidance and convenience will accelerate the development of solutions and measures against many problems, thus creating a serious momentum for progress for humanity. In the future, ChatGPT is also anticipated to offer opportunities that extend beyond current scientific research:

Literature review will be conducted much faster and easier. Academicians will be able to do thorough researches and benefit from studies in other languages as well. As it saves time and energy for academicians when researching, coding, summarizing, translating, classifying, they will have more time to do more researches (C5). However, it is also noted that the impact of ChatGPT on traditional education and academic processes will be limited. In this sense, ChatGPT has an indirect effect on academic life (C8).

The research we conduct today will seem quite basic in the future. Most likely, much more advanced research with AI support will be conducted in the future using much larger data sets (C2).

## 5 Discussion

The impact of ChatGPT on academic life is an interesting topic among academics (e.g., 9, 10, 43, 44, 45]. The findings show that ChatGPT is conceptualized by academics as a new, potential technology that can shape the future and can generate text like a human, which is seen as a positive attribute. Academics believe that ChatGPT will play a supportive role in scientific research and education processes. It can assist academics in identifying and reviewing literature related to their research interests. This finding is consistent with previous studies that suggest ChatGPT can be useful for literature review in academic research [9, 10, 45, 46). According to academics, ChatGPT can provide language support to researchers to understand literature in different languages and/or review their own translations for errors. This advantage is also recognized in previous studies as one of the academic benefits of ChatGPT [9,10,45]. Academics suggest that ChatGPT can also be used as an educational tool to facilitate access to sources and information for teaching purposes. Islam and Islam [10] also acknowledge the contribution of ChatGPT in this regard. Therefore, academics consider ChatGPT as a tool that can make their professional lives easier, save time and increase efficiency [[1], [2], [3], [4],^10^,^23^,^37^,[44], [45], [46], [47], [48], [49], [50], [51]].

Another promising finding is that academics believe ChatGPT has the potential to generate research ideas. Dergaa et al. [49] argue that ChatGPT can serve as an inspiration for academics to explore new topics and research areas. For academics, ChatGPT is an important technology to keep up with the fast-changing academic landscape (For a similar argument, see 10). In this context, our findings suggest that ChatGPT's contributions to the research process and idea generation can be described as a feature that enhances research quality. Indeed, a definition of ChatGPT as a technology with the potential to increase research productivity [47], enhance research output and improve the quality of academic publications has been made [45]. Despite its contributions, it is emphasized that ChatGPT's impact on the academic production process will be limited, and human judgment and interpretation are essential to idea development. As stated by Dönmez, Şahin, and Gülen [11], ChatGPT is a supportive tool in writing academic papers but is not capable of writing an entire article. It is not possible for ChatGPT to replace academics or human reasoning and interpretation [47,50].

In academic discussions of ChatGPT, two ethical issues have emerged as prominent concerns. The first is the fear of plagiarism, as highlighted by various academics [43,48,[52], [53], [54], [55], [56], [57], [58], [59]]. Concerns are raised over who owns the text generated by ChatGPT, and the possibility of researchers claiming authorship of text produced by ChatGPT as their own. This debate, which reveals the subject-object dichotomy of research, is paralleled by discussions on the recognition of ChatGPT's contribution to academic justice and the difficulty in determining research ownership [45,49]. The findings suggest that academics are worried about detecting plagiarism. It is known that software used to detect plagiarism in research texts generated by ChatGPT is inadequate (11, 60]. Another concern regarding plagiarism is that the use of ChatGPT in educational assessment processes may invalidate evaluations, as it becomes difficult to determine the authorship of assignments, raising concerns about student grades. This concern is reminiscent of the experiment in which ChatGPT passed a business exam [60]. The second ethical issue with ChatGPT is the concern about disinformation. Academics are skeptical about the accuracy and impartiality of the information obtained from ChatGPT. Risks associated with ChatGPT include biased information [10,45], which can make the content's reliability controversial [11], the potential to reflect and disseminate erroneous or biased information in the education process [9], the potential to generate scientific misinformation by creating misleading or incorrect content [61], the spread of disinformation and misinformation [10], the perpetuation of bias [47], and the potential for misusing concepts [49].

According to findings regarding the “future” ChatGPT's concerns about plagiarism are evident [53,54]. It is stated that detecting plagiarism will be a serious problem that will continue in the future. Alshater [47] also notes that in future research, the use of ChatGPT and similar technologies will require consistent ethical regulations. However, according to findings, academics indicate that ChatGPT will be able to provide opportunities for analyzing large amounts of data in the future. Other studies also support the idea that ChatGPT and similar technologies will enable the analysis of refined large datasets from numerous texts without human participation [10], even allowing for the analysis of large datasets without human participation. However, according to another finding, these types of opportunities may dull researchers' research skills. Islam and Islam [10] emphasize that students and academics may become dependent on AI applications, leading to weaknesses in productivity and critical thinking skills. Another future projection highlights the necessity of alternative teaching methods emphasized by academics. Other studies suggest that academics need to consider innovative ways in their teaching processes [43], by providing assignments that require critical thinking and problem-solving skills [49], and by rethinking assessment strategies and approaches in academic life [60].

## 6 Conclusion

The results revealed that ChatGPT could serve as a powerful assistant tool in scientific research and education and could also serve as a source of inspiration for new topics or research areas. However, the study also revealed that ChatGPT raises ethical concerns among academics, such as plagiarism and disinformation.

This study has some limitations. The study participants were from Turkey, a developed country located in Asia, which may not be representative of scholars from Western countries and other developing countries. In addition, most of the participants were male and aged between 30 and 39. Due to these limitations of the study, unequal distribution of gender, age and cultural variables may cause bias in the results.

Researchers can prepare scales in the future by using the conceptual framework of this research. In this way, generalisable studies with quantitative methodology and larger samples can be conducted with the use of statistical techniques. In addition, research diversity can be ensured by conducting interviews with culturally different communities and/or different age groups. However, since ChatGPT is still very new, the subject can be deepened with long-term observations or periodically repeated studies. Future studies can also focus on how AI, like ChatGPT, can be effectively utilized to analyze large amounts of data, looking at the benefits, challenges, and implications for academia.

## Ethical considerations

Ethical approval for this research was obtained from Akdeniz University Social Sciences and Humanities Scientific Research and Publication Ethics Committee (Approval ID: 07.03.2023-595804). The purpose of the study was explained to the participants, and a written informed consent was obtained from each respondent before administering the interviews. Confidentiality of information was maintained by omitting any personal identifier from the interviews. Collected data were also kept in a secure database without identifiers.

## Author contribution statement

All authors listed have significantly contributed to the development and the writing of this article. </p>

## Data availability statement

The data that has been used is confidential.

## Additional information

No additional information is available for this paper.

## Declaration of competing interest

The authors declare that they have no known competing financial interests or personal relationships that could have appeared to influence the work reported in this paper.
